# 4-Hy­droxy-3-[(4-hy­droxy-2-oxo-2*H*-3-chromen­yl)(3-thien­yl)meth­yl]-2*H*-chromen-2-one

**DOI:** 10.1107/S1600536811011676

**Published:** 2011-04-07

**Authors:** Mohammad Asad, Chuan-Wei Oo, Hasnah Osman, Mohd Mustaqim Rosli, Hoong-Kun Fun

**Affiliations:** a School of Chemical Sciences, Universiti Sains Malaysia, 11800 USM, Penang, Malaysia; b X-ray Crystallography Unit, School of Physics, Universiti Sains Malaysia, 11800 USM, Penang, Malaysia

## Abstract

The whole mol­ecule of the title compound, C_23_H_14_O_6_S, is disordered over two sets of sites with refined occupancies of 0.8733 (12):0.1267 (12). The dihedral angle between the mean planes through the chromene ring systems is 56.31 (5) and 55.2 (3)° for the major and minor components, respectively. In both components, a pair of intra­molecular O—H⋯O inter­actions generate rings of *S*(8) graph-set motif. In the crystal, the mol­ecules are linked by inter­molecular C—H⋯O inter­actions, forming chains along the *b* axis. The structure is further stabilized by π–π inter­actions with centroid–centroid distances of 3.594 (2) and 3.608 (5) Å.

## Related literature

For the biological activity of 4-hy­droxy­coumarins, see: Abdelhafez *et al.* (2010 ▶); Huang *et al.* (2010 ▶); Jacquot *et al.* (2001 ▶); Kokil *et al.* (2010 ▶); Siddiqui & Asad (2010 ▶); Skulnick *et al.* (1997 ▶); Sullivan *et al.* (1943 ▶). For hydrogen-bond motifs, see: Bernstein *et al.* (1995 ▶).
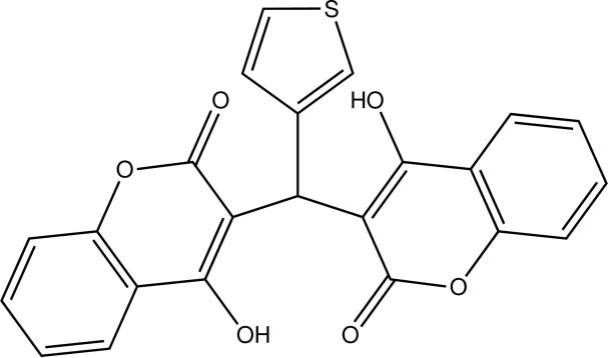


## Experimental

### 

#### Crystal data

C_23_H_14_O_6_S
*M* *_r_* = 418.40
Monoclinic, 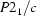

*a* = 7.8225 (2) Å
*b* = 8.9426 (2) Å
*c* = 27.1317 (6) Å
β = 100.679 (1)°
*V* = 1865.09 (8) Å^3^
*Z* = 4
Mo *K*α radiation
μ = 0.22 mm^−1^
*T* = 297 K
0.49 × 0.44 × 0.16 mm

#### Data collection

Bruker SMART APEXII CCD area-detector diffractometer
Absorption correction: multi-scan (*SADABS*; Bruker, 2009 ▶) *T*_min_ = 0.902, *T*_max_ = 0.966
20839 measured reflections
5420 independent reflections
3497 reflections with *I* > 2σ(*I*)
*R*_int_ = 0.031

#### Refinement

*R*[*F* ^2^ > 2σ(*F* ^2^)] = 0.044
*wR*(*F* ^2^) = 0.106
*S* = 1.02
5420 reflections
524 parameters
252 restraints
H-atom parameters constrained
Δρ_max_ = 0.17 e Å^−3^
Δρ_min_ = −0.21 e Å^−3^

### 

Data collection: *APEX2* (Bruker, 2009 ▶); cell refinement: *SAINT* (Bruker, 2009 ▶); data reduction: *SAINT*; program(s) used to solve structure: *SHELXTL* (Sheldrick, 2008 ▶); program(s) used to refine structure: *SHELXTL*; molecular graphics: *SHELXTL*; software used to prepare material for publication: *SHELXTL* and *PLATON* (Spek, 2009 ▶).

## Supplementary Material

Crystal structure: contains datablocks global, I. DOI: 10.1107/S1600536811011676/rz2575sup1.cif

Structure factors: contains datablocks I. DOI: 10.1107/S1600536811011676/rz2575Isup2.hkl

Additional supplementary materials:  crystallographic information; 3D view; checkCIF report

## Figures and Tables

**Table 1:** Hydrogen-bond geometry (Å, °)

*D*—H⋯*A*	*D*—H	H⋯*A*	*D*⋯*A*	*D*—H⋯*A*
O3—H3*B*⋯O4	0.82	1.89	2.7049 (19)	180
O6—H6*B*⋯O5	0.82	1.79	2.612 (2)	179
C21—H21*A*⋯O4^i^	0.93	2.56	3.475 (8)	168
O3*X*—H3*XB*⋯O4*X*	0.82	1.86	2.682 (15)	178
O6*X*—H6*XB*⋯O5*X*	0.82	1.69	2.49 (2)	168
C23*X*—H23*B*⋯O3*X*^i^	0.93	2.53	3.31 (5)	142

Symmetry code: (i) 
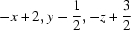
.

**Fig. 1.**
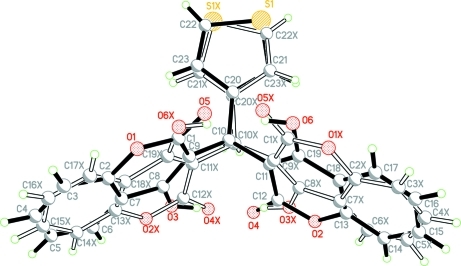
The molecular structure of the title compound showing a whole molecule disordered over two orientations.

**Fig. 2.**
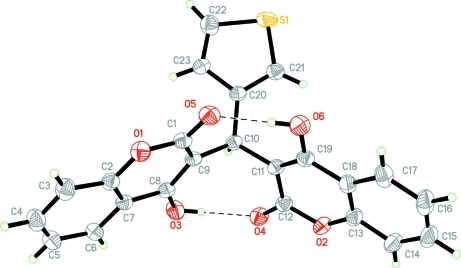
The molecular structure of the major component of the title compund, showing 30% probability displacement ellipsoids. Intramolecular hydrogen bonds are shown as dashed lines.

**Fig. 3.**
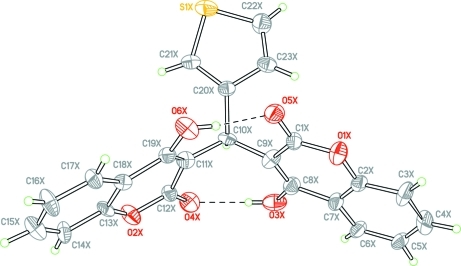
The molecular structure of the minor component of the title compund, showing 30% probability displacement ellipsoids. Intramolecular hydrogen bonds are shown as dashed lines.

**Fig. 4.**
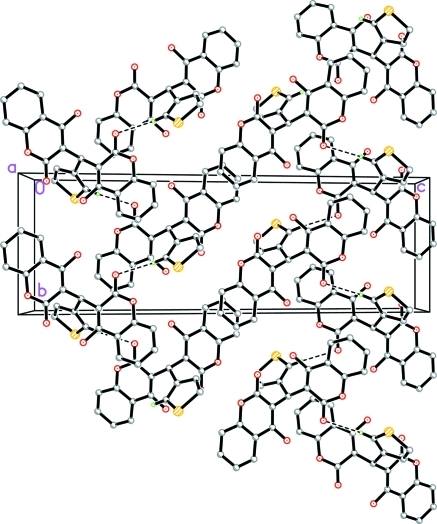
The crystal packing of the title compound viewed along the c axis. H atoms not involved in the hydrogen bonding interactions (dashed lines) are omitted for clarity. Only the major component of disorder is shown.

## supplementary crystallographic information

### Comment

The colourless crystalline bis 4-hydroxycoumarins are obtained from the condensation of coumarin and aldehyde derivatives (Sullivan *et al.*, 1943). A large number of structurally novel 4-hydroxycoumarin derivatives have exhibited estrogenic activity on MCF-7 breast carcinoma cells (Jacquot *et al.*, 2001) and possess several types of biological activities such as antifungal (Kokil *et al.*, 2010), anticoagulant (Abdelhafez *et al.*, 2010) anticancer (Huang *et al.*, 2010), anti-HIV (Skulnick *et al.*, 1997), antibacterial and anti-inflammatory (Siddiqui & Asad, 2010) activities.

In the title compound (Fig. 1), the whole molecule is disordered over two orientations with refined site occupancies of 0.873 (1):0.127 (1). The chromene ring systems are almost planar, with maximum deviation of 0.099 (3) Å for atom C9A (major component, Fig. 2) and 0.12 (2) Å for atom C11X (minor component, Fig. 3). The dihedral angles they form are 56.31 (5) and 55.2 (3)° for the major and minor components, respectively. Two pairs of intramolecular hydrogen bonds, O3—H3B···O4, O6—H6B···O5 (major component) and O3X—H3XB···O4X, O6X—H6XB···O5X (minor component), generate rings of S(8) graph-set motifs (Table 1; Bernstein *et al.*, 1995). In the crystal packing, intermolecular C—H···O hydrogen interactions (Table 1) link the molecules into chains along the *b* axis (Fig. 4). π—π stacking interactions with centroid-to-centroid distances of 3.594 (2) (major component) and 3.608 (5) Å (minor component) are also observed.

### Experimental

To a well-stirred solution of 4-hydroxycoumarin (6.17 mmol, 1.0 g) in ethanol (10 ml) containing two drops of acetic acid, thiophene-3-carboxaldehyde (3.1 mmol, 0.345 g) was added. The reaction mixture was stirred at room temperature for 8 hr. The completion of the reaction was monitored by TLC. After the reaction was completed, the crystallized solid was separated out, filtered, washed with ethanol and dried. The isolated product was further purified by recrystallization from a chloroform / methanol mixture (1:1 *v*/*v*) to give the pure title compound in 70% yield.

### Refinement

The whole molecule is disordered over two positions with refined occupancy ratio of 0.873 (1):0.127 (1). The same *U*_ij_ parameters were applied to the S1/S1X, C20/C20X and C22/C22X pairs. Distance and rigid body restrain were used. All H atoms were positioned geometrically and refined using a riding model, with O—H = 0.82Å,  C—H = 0.93–0.98Å, and with *U*_iso_ = 1.2 *U*_eq_(C) or 1.5 *U*_eq_(O).

### Crystal data

**Table:**

C_23_H_14_O_6_S	*F*(000) = 864
*M**_r_* = 418.40	*D*_x_ = 1.490 Mg m^−^^3^
Monoclinic, *P*2_1_/*c*	Mo *K*α radiation, λ = 0.71073 Å
Hall symbol: -P 2ybc	Cell parameters from 5379 reflections
*a* = 7.8225 (2) Å	θ = 2.4–26.0°
*b* = 8.9426 (2) Å	µ = 0.22 mm^−^^1^
*c* = 27.1317 (6) Å	*T* = 297 K
β = 100.679 (1)°	Plate, colourless
*V* = 1865.09 (8) Å^3^	0.49 × 0.44 × 0.16 mm
*Z* = 4	

### Data collection

**Table:**

Bruker SMART APEXII CCD area-detector diffractometer	5420 independent reflections
Radiation source: fine-focus sealed tube	3497 reflections with *I* > 2σ(*I*)
graphite	*R*_int_ = 0.031
φ and ω scans	θ_max_ = 30.0°, θ_min_ = 1.5°
Absorption correction: multi-scan (*SADABS*; Bruker, 2009)	*h* = −11→9
*T*_min_ = 0.902, *T*_max_ = 0.966	*k* = −12→12
20839 measured reflections	*l* = −36→38

### Refinement

**Table:**

Refinement on *F*^2^	Primary atom site location: structure-invariant direct methods
Least-squares matrix: full	Secondary atom site location: difference Fourier map
*R*[*F*^2^ > 2σ(*F*^2^)] = 0.044	Hydrogen site location: inferred from neighbouring sites
*wR*(*F*^2^) = 0.106	H-atom parameters constrained
*S* = 1.02	*w* = 1/[σ^2^(*F*_o_^2^) + (0.0409*P*)^2^ + 0.2504*P*] where *P* = (*F*_o_^2^ + 2*F*_c_^2^)/3
5420 reflections	(Δ/σ)_max_ = 0.001
524 parameters	Δρ_max_ = 0.17 e Å^−^^3^
252 restraints	Δρ_min_ = −0.21 e Å^−^^3^

### Special details

**Table:**

Geometry. All esds (except the esd in the dihedral angle between two l.s. planes) are estimated using the full covariance matrix. The cell esds are taken into account individually in the estimation of esds in distances, angles and torsion angles; correlations between esds in cell parameters are only used when they are defined by crystal symmetry. An approximate (isotropic) treatment of cell esds is used for estimating esds involving l.s. planes.
Refinement. Refinement of F^2^ against ALL reflections. The weighted R-factor wR and goodness of fit S are based on F^2^, conventional R-factors R are based on F, with F set to zero for negative F^2^. The threshold expression of F^2^ > 2sigma(F^2^) is used only for calculating R-factors(gt) etc. and is not relevant to the choice of reflections for refinement. R-factors based on F^2^ are statistically about twice as large as those based on F, and R- factors based on ALL data will be even larger.

### Fractional atomic coordinates and isotropic or equivalent isotropic displacement parameters (Å^2^)

**Table:**

	*x*	*y*	*z*	*U*_iso_*/*U*_eq_	Occ. (<1)
S1	1.45953 (7)	−0.16107 (8)	0.88120 (2)	0.06222 (18)	0.8733 (12)
O1	0.84520 (16)	0.13801 (13)	0.97160 (4)	0.0513 (3)	0.8733 (12)
O2	0.76016 (19)	0.09511 (15)	0.72666 (5)	0.0575 (3)	0.8733 (12)
O3	0.9259 (3)	0.42341 (16)	0.85963 (6)	0.0587 (5)	0.8733 (12)
H3B	0.9165	0.3803	0.8326	0.088*	0.8733 (12)
O4	0.89510 (17)	0.28109 (14)	0.77045 (4)	0.0553 (3)	0.8733 (12)
O5	0.91759 (18)	−0.05801 (15)	0.93159 (5)	0.0536 (3)	0.8733 (12)
O6	0.8790 (3)	−0.19519 (16)	0.84525 (7)	0.0568 (5)	0.8733 (12)
H6B	0.8911	−0.1515	0.8722	0.085*	0.8733 (12)
C1	0.9078 (6)	0.0795 (4)	0.93266 (16)	0.0437 (6)	0.8733 (12)
C2	0.8154 (3)	0.2889 (2)	0.97464 (11)	0.0454 (5)	0.8733 (12)
C3	0.7596 (4)	0.3388 (4)	1.01761 (10)	0.0595 (7)	0.8733 (12)
H3A	0.7469	0.2730	1.0432	0.071*	0.8733 (12)
C4	0.7237 (4)	0.4894 (4)	1.02097 (11)	0.0656 (7)	0.8733 (12)
H4A	0.6850	0.5251	1.0492	0.079*	0.8733 (12)
C5	0.7441 (4)	0.5881 (3)	0.98318 (13)	0.0642 (7)	0.8733 (12)
H5A	0.7203	0.6892	0.9864	0.077*	0.8733 (12)
C6	0.7992 (6)	0.5377 (3)	0.94110 (12)	0.0554 (6)	0.8733 (12)
H6A	0.8108	0.6040	0.9155	0.066*	0.8733 (12)
C7	0.8381 (4)	0.3853 (3)	0.93660 (9)	0.0428 (5)	0.8733 (12)
C8	0.9078 (2)	0.32467 (17)	0.89533 (6)	0.0420 (4)	0.8733 (12)
C9	0.9528 (4)	0.1773 (2)	0.89474 (9)	0.0378 (4)	0.8733 (12)
C10	1.0443 (4)	0.1109 (4)	0.85510 (13)	0.0396 (8)	0.8733 (12)
H10A	1.0938	0.1980	0.8409	0.048*	0.8733 (12)
C11	0.9188 (6)	0.0448 (3)	0.81078 (11)	0.0407 (6)	0.8733 (12)
C12	0.8608 (3)	0.1469 (3)	0.76989 (8)	0.0461 (5)	0.8733 (12)
C13	0.7010 (9)	−0.0501 (5)	0.72286 (15)	0.0534 (7)	0.8733 (12)
C14	0.5957 (4)	−0.0905 (4)	0.67758 (12)	0.0706 (8)	0.8733 (12)
H14A	0.5711	−0.0229	0.6512	0.085*	0.8733 (12)
C15	0.5291 (6)	−0.2335 (5)	0.67314 (16)	0.0771 (12)	0.8733 (12)
H15A	0.4580	−0.2631	0.6434	0.093*	0.8733 (12)
C16	0.5671 (5)	−0.3337 (4)	0.71256 (16)	0.0723 (9)	0.8733 (12)
H16A	0.5216	−0.4300	0.7088	0.087*	0.8733 (12)
C17	0.6707 (3)	−0.2930 (3)	0.75702 (11)	0.0623 (6)	0.8733 (12)
H17A	0.6953	−0.3614	0.7832	0.075*	0.8733 (12)
C18	0.7395 (4)	−0.1476 (3)	0.76278 (10)	0.0497 (6)	0.8733 (12)
C19	0.8497 (3)	−0.0960 (2)	0.80817 (8)	0.0447 (4)	0.8733 (12)
C20	1.2020 (10)	0.0152 (9)	0.8759 (4)	0.0407 (7)	0.8733 (12)
C21	1.2656 (6)	−0.0971 (7)	0.8509 (3)	0.0486 (12)	0.8733 (12)
H21A	1.2083	−0.1347	0.8203	0.058*	0.8733 (12)
C22	1.4673 (6)	−0.0292 (5)	0.93173 (17)	0.0635 (9)	0.8733 (12)
H22A	1.5562	−0.0180	0.9594	0.076*	0.8733 (12)
C23	1.3173 (5)	0.0507 (4)	0.92124 (15)	0.0470 (9)	0.8733 (12)
H23A	1.2913	0.1249	0.9427	0.056*	0.8733 (12)
S1X	1.4837 (13)	−0.0494 (11)	0.9255 (3)	0.06222 (18)	0.1267 (12)
O1X	0.7724 (12)	−0.2372 (9)	0.8067 (3)	0.062 (2)	0.1267 (12)
O2X	0.8652 (14)	0.4728 (9)	0.8836 (4)	0.062 (2)	0.1267 (12)
O3X	0.8690 (18)	0.1683 (14)	0.7452 (5)	0.072 (4)	0.1267 (12)
H3XB	0.8894	0.2340	0.7666	0.107*	0.1267 (12)
O4X	0.9408 (13)	0.3872 (9)	0.8135 (3)	0.063 (2)	0.1267 (12)
O5X	0.9168 (13)	−0.1677 (9)	0.8805 (4)	0.057 (2)	0.1267 (12)
O6X	0.921 (3)	0.041 (2)	0.9416 (9)	0.060 (5)	0.1267 (12)
H6XB	0.9180	−0.0187	0.9186	0.089*	0.1267 (12)
C1X	0.867 (2)	−0.1342 (16)	0.8351 (5)	0.044 (3)	0.1267 (12)
C2X	0.708 (2)	−0.205 (2)	0.7575 (5)	0.041 (3)	0.1267 (12)
C3X	0.612 (4)	−0.319 (2)	0.7318 (8)	0.067 (5)	0.1267 (12)
H3XA	0.5931	−0.4090	0.7469	0.081*	0.1267 (12)
C4X	0.547 (4)	−0.289 (2)	0.6821 (8)	0.063 (6)	0.1267 (12)
H4XA	0.4900	−0.3648	0.6620	0.075*	0.1267 (12)
C5X	0.564 (3)	−0.150 (2)	0.6611 (7)	0.062 (4)	0.1267 (12)
H5XA	0.5047	−0.1309	0.6287	0.074*	0.1267 (12)
C6X	0.662 (2)	−0.041 (2)	0.6862 (7)	0.057 (4)	0.1267 (12)
H6XA	0.6848	0.0477	0.6706	0.068*	0.1267 (12)
C7X	0.730 (6)	−0.067 (3)	0.7375 (9)	0.053 (5)	0.1267 (12)
C8X	0.8470 (16)	0.0409 (14)	0.7668 (4)	0.051 (3)	0.1267 (12)
C9X	0.903 (4)	0.007 (2)	0.8152 (6)	0.044 (4)	0.1267 (12)
C10X	1.036 (3)	0.109 (2)	0.8465 (9)	0.035 (3)	0.1267 (12)
H10B	1.0791	0.1734	0.8222	0.042*	0.1267 (12)
C11X	0.964 (3)	0.2153 (14)	0.8809 (6)	0.044 (3)	0.1267 (12)
C12X	0.924 (2)	0.3609 (12)	0.8574 (5)	0.047 (3)	0.1267 (12)
C13X	0.829 (3)	0.4443 (15)	0.9315 (6)	0.038 (3)	0.1267 (12)
C14X	0.774 (4)	0.565 (2)	0.9559 (8)	0.053 (5)	0.1267 (12)
H14B	0.7633	0.6592	0.9412	0.064*	0.1267 (12)
C15X	0.737 (3)	0.5410 (17)	1.0020 (8)	0.054 (6)	0.1267 (12)
H15B	0.7079	0.6225	1.0200	0.065*	0.1267 (12)
C16X	0.741 (3)	0.4021 (18)	1.0232 (7)	0.062 (6)	0.1267 (12)
H16B	0.7078	0.3873	1.0540	0.074*	0.1267 (12)
C17X	0.796 (3)	0.2868 (17)	0.9977 (6)	0.047 (4)	0.1267 (12)
H17B	0.8025	0.1920	1.0120	0.057*	0.1267 (12)
C18X	0.8430 (18)	0.3042 (14)	0.9506 (6)	0.037 (3)	0.1267 (12)
C19X	0.9143 (15)	0.1805 (13)	0.9233 (5)	0.042 (2)	0.1267 (12)
C20X	1.196 (7)	0.025 (7)	0.873 (3)	0.0407 (7)	0.1267 (12)
C21X	1.319 (3)	0.076 (3)	0.9116 (10)	0.037 (4)	0.1267 (12)
H21B	1.3146	0.1672	0.9278	0.044*	0.1267 (12)
C22X	1.411 (2)	−0.149 (2)	0.8693 (7)	0.0635 (9)	0.1267 (12)
H22B	1.4762	−0.2128	0.8532	0.076*	0.1267 (12)
C23X	1.242 (4)	−0.114 (5)	0.8543 (18)	0.047 (6)	0.1267 (12)
H23B	1.1634	−0.1750	0.8336	0.057*	0.1267 (12)

### Atomic displacement parameters (Å^2^)

**Table:**

	*U*^11^	*U*^22^	*U*^33^	*U*^12^	*U*^13^	*U*^23^
S1	0.0455 (3)	0.0719 (3)	0.0698 (4)	0.0188 (3)	0.0120 (2)	0.0105 (3)
O1	0.0552 (8)	0.0543 (7)	0.0469 (7)	−0.0024 (6)	0.0156 (6)	0.0068 (5)
O2	0.0571 (8)	0.0671 (8)	0.0436 (7)	0.0022 (7)	−0.0034 (6)	−0.0004 (6)
O3	0.0849 (12)	0.0401 (8)	0.0535 (9)	0.0056 (10)	0.0192 (8)	0.0106 (8)
O4	0.0653 (9)	0.0515 (7)	0.0476 (7)	−0.0016 (6)	0.0069 (6)	0.0115 (6)
O5	0.0626 (9)	0.0421 (6)	0.0557 (8)	−0.0001 (7)	0.0100 (6)	0.0122 (6)
O6	0.0620 (10)	0.0443 (9)	0.0625 (11)	−0.0074 (9)	0.0068 (8)	0.0063 (8)
C1	0.0369 (11)	0.0434 (16)	0.0490 (15)	−0.0004 (13)	0.0036 (10)	0.0047 (12)
C2	0.0351 (13)	0.0552 (11)	0.0458 (19)	−0.0018 (9)	0.0069 (14)	−0.0021 (12)
C3	0.0526 (15)	0.074 (2)	0.0549 (15)	−0.0058 (18)	0.0174 (11)	−0.0067 (15)
C4	0.0537 (14)	0.081 (2)	0.0664 (17)	−0.0012 (18)	0.0211 (13)	−0.0181 (16)
C5	0.0624 (17)	0.0629 (16)	0.066 (2)	0.0120 (15)	0.0087 (16)	−0.0126 (14)
C6	0.0563 (16)	0.0508 (15)	0.058 (2)	0.0082 (13)	0.0068 (16)	−0.0037 (13)
C7	0.0374 (10)	0.0428 (15)	0.0464 (12)	0.0009 (16)	0.0029 (9)	0.0018 (11)
C8	0.0404 (9)	0.0421 (8)	0.0422 (9)	0.0003 (7)	0.0044 (7)	0.0050 (7)
C9	0.0321 (10)	0.0401 (11)	0.0405 (13)	−0.0009 (10)	0.0048 (9)	0.0053 (9)
C10	0.0350 (14)	0.0411 (13)	0.0414 (15)	−0.0036 (9)	0.0034 (10)	0.0042 (11)
C11	0.0325 (14)	0.0473 (12)	0.0423 (12)	−0.0031 (10)	0.0067 (11)	0.0021 (9)
C12	0.0412 (10)	0.0550 (13)	0.0421 (12)	0.0008 (9)	0.0077 (10)	0.0008 (10)
C13	0.041 (2)	0.0665 (15)	0.051 (2)	0.0010 (14)	0.0069 (18)	−0.0136 (14)
C14	0.051 (2)	0.093 (3)	0.0623 (18)	0.0062 (17)	−0.0046 (14)	−0.0223 (15)
C15	0.0396 (16)	0.106 (3)	0.083 (2)	−0.004 (2)	0.0024 (15)	−0.046 (2)
C16	0.046 (2)	0.0877 (18)	0.088 (3)	−0.0199 (13)	0.023 (2)	−0.0410 (19)
C17	0.0452 (15)	0.0625 (14)	0.0822 (18)	−0.0150 (11)	0.0201 (12)	−0.0222 (13)
C18	0.0351 (14)	0.0551 (16)	0.0600 (14)	−0.0031 (12)	0.0118 (10)	−0.0122 (12)
C19	0.0356 (10)	0.0474 (10)	0.0515 (11)	0.0002 (8)	0.0091 (9)	0.0009 (9)
C20	0.0356 (10)	0.0425 (15)	0.0438 (15)	−0.0002 (7)	0.0070 (7)	0.0053 (14)
C21	0.0420 (16)	0.0546 (18)	0.0502 (16)	0.0024 (13)	0.0109 (14)	0.0014 (16)
C22	0.0570 (19)	0.069 (2)	0.0671 (18)	−0.0021 (13)	0.0172 (12)	−0.0084 (12)
C23	0.0445 (13)	0.0495 (16)	0.0445 (17)	−0.0007 (11)	0.0016 (12)	0.0047 (14)
S1X	0.0455 (3)	0.0719 (3)	0.0698 (4)	0.0188 (3)	0.0120 (2)	0.0105 (3)
O1X	0.063 (6)	0.060 (5)	0.062 (5)	−0.018 (4)	0.008 (4)	−0.010 (4)
O2X	0.076 (7)	0.044 (5)	0.070 (6)	0.015 (4)	0.025 (5)	0.003 (4)
O3X	0.067 (8)	0.079 (7)	0.066 (8)	0.017 (6)	0.002 (7)	0.027 (7)
O4X	0.073 (6)	0.048 (5)	0.069 (5)	−0.004 (4)	0.018 (5)	0.011 (4)
O5X	0.064 (6)	0.045 (5)	0.057 (5)	−0.001 (4)	−0.003 (5)	0.008 (4)
O6X	0.064 (10)	0.049 (8)	0.074 (11)	0.003 (8)	0.033 (9)	0.009 (6)
C1X	0.040 (7)	0.045 (8)	0.047 (7)	−0.001 (8)	0.006 (7)	−0.011 (6)
C2X	0.025 (8)	0.054 (10)	0.046 (6)	−0.005 (8)	0.010 (5)	−0.010 (7)
C3X	0.057 (17)	0.083 (11)	0.060 (11)	−0.026 (8)	0.005 (10)	−0.026 (9)
C4X	0.072 (15)	0.081 (12)	0.045 (8)	−0.007 (12)	0.036 (9)	−0.034 (8)
C5X	0.030 (9)	0.076 (14)	0.074 (11)	−0.004 (8)	−0.003 (8)	−0.022 (8)
C6X	0.042 (10)	0.075 (10)	0.048 (8)	0.001 (7)	−0.007 (7)	−0.018 (7)
C7X	0.047 (16)	0.063 (10)	0.047 (11)	0.001 (12)	0.002 (12)	−0.021 (7)
C8X	0.045 (7)	0.054 (7)	0.054 (6)	−0.006 (5)	0.007 (5)	−0.005 (5)
C9X	0.037 (8)	0.068 (11)	0.031 (6)	−0.014 (10)	0.014 (5)	0.004 (5)
C10X	0.042 (9)	0.032 (7)	0.034 (7)	0.011 (5)	0.012 (5)	0.022 (5)
C11X	0.043 (7)	0.035 (6)	0.055 (9)	−0.008 (6)	0.010 (7)	0.009 (4)
C12X	0.046 (7)	0.031 (6)	0.062 (7)	0.000 (7)	0.004 (6)	−0.006 (5)
C13X	0.039 (7)	0.019 (6)	0.060 (7)	0.005 (8)	0.016 (6)	0.005 (6)
C14X	0.082 (18)	0.038 (7)	0.040 (11)	0.002 (8)	0.011 (9)	−0.010 (7)
C15X	0.063 (10)	0.035 (7)	0.080 (15)	−0.005 (8)	0.051 (13)	0.004 (7)
C16X	0.099 (15)	0.023 (8)	0.073 (10)	−0.001 (10)	0.045 (9)	−0.002 (7)
C17X	0.052 (9)	0.046 (7)	0.047 (12)	0.006 (6)	0.018 (10)	−0.005 (7)
C18X	0.031 (6)	0.038 (6)	0.045 (8)	−0.003 (6)	0.011 (6)	0.007 (5)
C19X	0.037 (6)	0.040 (6)	0.048 (7)	0.002 (5)	0.003 (5)	0.000 (5)
C20X	0.0356 (10)	0.0425 (15)	0.0438 (15)	−0.0002 (7)	0.0070 (7)	0.0053 (14)
C21X	0.037 (7)	0.035 (6)	0.041 (9)	−0.003 (5)	0.011 (5)	0.024 (5)
C22X	0.0570 (19)	0.069 (2)	0.0671 (18)	−0.0021 (13)	0.0172 (12)	−0.0084 (12)
C23X	0.039 (8)	0.047 (9)	0.058 (11)	0.004 (8)	0.015 (9)	0.009 (7)

### Geometric parameters (Å, °)

**Table:**

S1—C21	1.686 (4)	S1X—C21X	1.696 (18)
S1—C22	1.801 (3)	S1X—C22X	1.767 (15)
O1—C1	1.350 (5)	O1X—C1X	1.335 (14)
O1—C2	1.374 (2)	O1X—C2X	1.369 (13)
O2—C12	1.367 (3)	O2X—C12X	1.359 (12)
O2—C13	1.376 (3)	O2X—C13X	1.403 (14)
O3—C8	1.337 (2)	O3X—C8X	1.306 (13)
O3—H3B	0.8200	O3X—H3XB	0.8200
O4—C12	1.230 (3)	O4X—C12X	1.243 (14)
O5—C1	1.232 (3)	O5X—C1X	1.256 (14)
O6—C19	1.329 (2)	O6X—C19X	1.337 (13)
O6—H6B	0.8200	O6X—H6XB	0.8200
C1—C9	1.443 (4)	C1X—C9X	1.422 (15)
C2—C7	1.381 (3)	C2X—C7X	1.368 (16)
C2—C3	1.392 (3)	C2X—C3X	1.375 (15)
C3—C4	1.382 (4)	C3X—C4X	1.378 (17)
C3—H3A	0.9300	C3X—H3XA	0.9300
C4—C5	1.385 (4)	C4X—C5X	1.385 (18)
C4—H4A	0.9300	C4X—H4XA	0.9300
C5—C6	1.369 (3)	C5X—C6X	1.348 (15)
C5—H5A	0.9300	C5X—H5XA	0.9300
C6—C7	1.407 (3)	C6X—C7X	1.416 (15)
C6—H6A	0.9300	C6X—H6XA	0.9300
C7—C8	1.439 (3)	C7X—C8X	1.459 (15)
C8—C9	1.365 (2)	C8X—C9X	1.341 (15)
C9—C10	1.519 (4)	C9X—C10X	1.522 (17)
C10—C20	1.521 (3)	C10X—C11X	1.511 (17)
C10—C11	1.524 (3)	C10X—C20X	1.520 (17)
C10—H10A	0.9800	C10X—H10B	0.9800
C11—C19	1.368 (3)	C11X—C19X	1.320 (13)
C11—C12	1.443 (3)	C11X—C12X	1.457 (14)
C13—C18	1.380 (4)	C13X—C18X	1.352 (12)
C13—C14	1.394 (4)	C13X—C14X	1.374 (14)
C14—C15	1.378 (5)	C14X—C15X	1.352 (16)
C14—H14A	0.9300	C14X—H14B	0.9300
C15—C16	1.385 (5)	C15X—C16X	1.367 (15)
C15—H15A	0.9300	C15X—H15B	0.9300
C16—C17	1.372 (4)	C16X—C17X	1.355 (14)
C16—H16A	0.9300	C16X—H16B	0.9300
C17—C18	1.405 (3)	C17X—C18X	1.400 (14)
C17—H17A	0.9300	C17X—H17B	0.9300
C18—C19	1.442 (3)	C18X—C19X	1.496 (13)
C20—C21	1.356 (6)	C20X—C21X	1.36 (2)
C20—C23	1.420 (5)	C20X—C23X	1.42 (2)
C21—H21A	0.9300	C21X—H21B	0.9300
C22—C23	1.358 (5)	C22X—C23X	1.34 (2)
C22—H22A	0.9300	C22X—H22B	0.9300
C23—H23A	0.9300	C23X—H23B	0.9300
			
C21—S1—C22	92.8 (2)	C21X—S1X—C22X	91.7 (11)
C1—O1—C2	121.19 (19)	C1X—O1X—C2X	118.8 (11)
C12—O2—C13	121.07 (19)	C12X—O2X—C13X	119.8 (9)
C8—O3—H3B	109.5	C8X—O3X—H3XB	109.5
C19—O6—H6B	109.5	C19X—O6X—H6XB	109.5
O5—C1—O1	116.0 (3)	O5X—C1X—O1X	116.0 (13)
O5—C1—C9	124.3 (4)	O5X—C1X—C9X	122.6 (11)
O1—C1—C9	119.6 (3)	O1X—C1X—C9X	121.4 (13)
O1—C2—C7	121.4 (3)	C7X—C2X—O1X	121.7 (15)
O1—C2—C3	116.6 (3)	C7X—C2X—C3X	124.3 (13)
C7—C2—C3	122.0 (2)	O1X—C2X—C3X	113.9 (15)
C4—C3—C2	117.8 (3)	C2X—C3X—C4X	114.9 (16)
C4—C3—H3A	121.1	C2X—C3X—H3XA	122.5
C2—C3—H3A	121.1	C4X—C3X—H3XA	122.5
C3—C4—C5	121.3 (3)	C3X—C4X—C5X	122.0 (18)
C3—C4—H4A	119.4	C3X—C4X—H4XA	119.0
C5—C4—H4A	119.4	C5X—C4X—H4XA	119.0
C6—C5—C4	120.3 (3)	C6X—C5X—C4X	122.1 (18)
C6—C5—H5A	119.8	C6X—C5X—H5XA	119.0
C4—C5—H5A	119.8	C4X—C5X—H5XA	119.0
C5—C6—C7	119.9 (3)	C5X—C6X—C7X	116.9 (16)
C5—C6—H6A	120.1	C5X—C6X—H6XA	121.6
C7—C6—H6A	120.1	C7X—C6X—H6XA	121.6
C2—C7—C6	118.7 (2)	C2X—C7X—C6X	119.2 (14)
C2—C7—C8	117.8 (2)	C2X—C7X—C8X	119.6 (15)
C6—C7—C8	123.5 (2)	C6X—C7X—C8X	120.5 (16)
O3—C8—C9	124.53 (17)	O3X—C8X—C9X	126.1 (13)
O3—C8—C7	114.90 (17)	O3X—C8X—C7X	117.1 (12)
C9—C8—C7	120.56 (18)	C9X—C8X—C7X	116.2 (12)
C8—C9—C1	118.7 (3)	C8X—C9X—C1X	121.5 (14)
C8—C9—C10	122.8 (2)	C8X—C9X—C10X	118.5 (14)
C1—C9—C10	118.5 (3)	C1X—C9X—C10X	118.8 (15)
C9—C10—C20	114.5 (5)	C11X—C10X—C20X	112 (2)
C9—C10—C11	113.0 (3)	C11X—C10X—C9X	115.0 (19)
C20—C10—C11	115.7 (3)	C20X—C10X—C9X	113 (2)
C9—C10—H10A	103.9	C11X—C10X—H10B	105.1
C20—C10—H10A	103.9	C20X—C10X—H10B	105.1
C11—C10—H10A	103.9	C9X—C10X—H10B	105.1
C19—C11—C12	118.5 (2)	C19X—C11X—C12X	121.3 (12)
C19—C11—C10	126.1 (3)	C19X—C11X—C10X	126.5 (14)
C12—C11—C10	115.2 (2)	C12X—C11X—C10X	111.4 (13)
O4—C12—O2	115.5 (2)	O4X—C12X—O2X	117.9 (11)
O4—C12—C11	124.8 (2)	O4X—C12X—C11X	122.5 (11)
O2—C12—C11	119.7 (2)	O2X—C12X—C11X	119.6 (11)
O2—C13—C18	121.1 (3)	C18X—C13X—C14X	123.4 (13)
O2—C13—C14	116.5 (3)	C18X—C13X—O2X	120.6 (12)
C18—C13—C14	122.4 (3)	C14X—C13X—O2X	115.9 (12)
C15—C14—C13	118.0 (3)	C15X—C14X—C13X	117.5 (15)
C15—C14—H14A	121.0	C15X—C14X—H14B	121.3
C13—C14—H14A	121.0	C13X—C14X—H14B	121.3
C14—C15—C16	120.6 (3)	C14X—C15X—C16X	122.7 (15)
C14—C15—H15A	119.7	C14X—C15X—H15B	118.7
C16—C15—H15A	119.7	C16X—C15X—H15B	118.7
C17—C16—C15	121.0 (3)	C17X—C16X—C15X	117.5 (15)
C17—C16—H16A	119.5	C17X—C16X—H16B	121.3
C15—C16—H16A	119.5	C15X—C16X—H16B	121.3
C16—C17—C18	119.7 (3)	C16X—C17X—C18X	122.8 (14)
C16—C17—H17A	120.2	C16X—C17X—H17B	118.6
C18—C17—H17A	120.2	C18X—C17X—H17B	118.6
C13—C18—C17	118.3 (3)	C13X—C18X—C17X	115.9 (12)
C13—C18—C19	118.5 (3)	C13X—C18X—C19X	120.3 (13)
C17—C18—C19	123.2 (3)	C17X—C18X—C19X	123.6 (13)
O6—C19—C11	124.4 (2)	C11X—C19X—O6X	123.0 (14)
O6—C19—C18	115.2 (2)	C11X—C19X—C18X	117.1 (11)
C11—C19—C18	120.5 (2)	O6X—C19X—C18X	119.8 (15)
C21—C20—C23	111.5 (3)	C21X—C20X—C23X	112.2 (15)
C21—C20—C10	125.4 (5)	C21X—C20X—C10X	127 (2)
C23—C20—C10	122.4 (3)	C23X—C20X—C10X	120 (2)
C20—C21—S1	112.8 (3)	C20X—C21X—S1X	110.6 (15)
C20—C21—H21A	123.6	C20X—C21X—H21B	124.7
S1—C21—H21A	123.6	S1X—C21X—H21B	124.7
C23—C22—S1	106.2 (3)	C23X—C22X—S1X	106.7 (17)
C23—C22—H22A	126.9	C23X—C22X—H22B	126.7
S1—C22—H22A	126.9	S1X—C22X—H22B	126.7
C22—C23—C20	116.7 (4)	C22X—C23X—C20X	113 (2)
C22—C23—H23A	121.7	C22X—C23X—H23B	123.4
C20—C23—H23A	121.7	C20X—C23X—H23B	123.4
			
C2—O1—C1—O5	−174.8 (3)	C2X—O1X—C1X—O5X	177.2 (14)
C2—O1—C1—C9	3.2 (5)	C2X—O1X—C1X—C9X	0(3)
C1—O1—C2—C7	4.0 (4)	C1X—O1X—C2X—C7X	−4(4)
C1—O1—C2—C3	−176.7 (3)	C1X—O1X—C2X—C3X	−180.0 (19)
O1—C2—C3—C4	−178.1 (2)	C7X—C2X—C3X—C4X	4(5)
C7—C2—C3—C4	1.2 (4)	O1X—C2X—C3X—C4X	−180 (2)
C2—C3—C4—C5	−0.7 (5)	C2X—C3X—C4X—C5X	−6(4)
C3—C4—C5—C6	0.7 (6)	C3X—C4X—C5X—C6X	8(4)
C4—C5—C6—C7	−1.1 (7)	C4X—C5X—C6X—C7X	−8(4)
O1—C2—C7—C6	177.6 (3)	O1X—C2X—C7X—C6X	179 (3)
C3—C2—C7—C6	−1.7 (4)	C3X—C2X—C7X—C6X	−5(6)
O1—C2—C7—C8	−4.9 (4)	O1X—C2X—C7X—C8X	9(6)
C3—C2—C7—C8	175.8 (2)	C3X—C2X—C7X—C8X	−175 (3)
C5—C6—C7—C2	1.6 (6)	C5X—C6X—C7X—C2X	6(6)
C5—C6—C7—C8	−175.7 (4)	C5X—C6X—C7X—C8X	176 (3)
C2—C7—C8—O3	179.7 (2)	C2X—C7X—C8X—O3X	177 (3)
C6—C7—C8—O3	−3.0 (4)	C6X—C7X—C8X—O3X	7(6)
C2—C7—C8—C9	−1.4 (4)	C2X—C7X—C8X—C9X	−11 (6)
C6—C7—C8—C9	175.9 (3)	C6X—C7X—C8X—C9X	179 (4)
O3—C8—C9—C1	−172.9 (3)	O3X—C8X—C9X—C1X	178 (2)
C7—C8—C9—C1	8.2 (4)	C7X—C8X—C9X—C1X	8(5)
O3—C8—C9—C10	5.5 (4)	O3X—C8X—C9X—C10X	−15 (4)
C7—C8—C9—C10	−173.3 (2)	C7X—C8X—C9X—C10X	175 (3)
O5—C1—C9—C8	168.5 (3)	O5X—C1X—C9X—C8X	−179 (2)
O1—C1—C9—C8	−9.3 (5)	O1X—C1X—C9X—C8X	−2(4)
O5—C1—C9—C10	−10.0 (6)	O5X—C1X—C9X—C10X	14 (4)
O1—C1—C9—C10	172.2 (3)	O1X—C1X—C9X—C10X	−170 (2)
C8—C9—C10—C20	131.6 (5)	C8X—C9X—C10X—C11X	102 (3)
C1—C9—C10—C20	−50.0 (6)	C1X—C9X—C10X—C11X	−90 (3)
C8—C9—C10—C11	−93.1 (4)	C8X—C9X—C10X—C20X	−127 (5)
C1—C9—C10—C11	85.3 (4)	C1X—C9X—C10X—C20X	41 (6)
C9—C10—C11—C19	−84.8 (5)	C20X—C10X—C11X—C19X	−56 (5)
C20—C10—C11—C19	49.9 (9)	C9X—C10X—C11X—C19X	75 (3)
C9—C10—C11—C12	90.6 (4)	C20X—C10X—C11X—C12X	134 (4)
C20—C10—C11—C12	−134.7 (7)	C9X—C10X—C11X—C12X	−95 (2)
C13—O2—C12—O4	−174.6 (4)	C13X—O2X—C12X—O4X	174.1 (16)
C13—O2—C12—C11	5.4 (5)	C13X—O2X—C12X—C11X	−5(3)
C19—C11—C12—O4	170.1 (3)	C19X—C11X—C12X—O4X	−166.6 (18)
C10—C11—C12—O4	−5.6 (5)	C10X—C11X—C12X—O4X	4(3)
C19—C11—C12—O2	−9.9 (6)	C19X—C11X—C12X—O2X	13 (3)
C10—C11—C12—O2	174.3 (3)	C10X—C11X—C12X—O2X	−177.1 (16)
C12—O2—C13—C18	0.8 (9)	C12X—O2X—C13X—C18X	−4(3)
C12—O2—C13—C14	178.4 (4)	C12X—O2X—C13X—C14X	178 (2)
O2—C13—C14—C15	−177.7 (5)	C18X—C13X—C14X—C15X	2(4)
C18—C13—C14—C15	−0.2 (9)	O2X—C13X—C14X—C15X	179 (2)
C13—C14—C15—C16	−0.3 (7)	C13X—C14X—C15X—C16X	−4(4)
C14—C15—C16—C17	0.4 (7)	C14X—C15X—C16X—C17X	4(4)
C15—C16—C17—C18	0.0 (6)	C15X—C16X—C17X—C18X	−2(4)
O2—C13—C18—C17	178.1 (5)	C14X—C13X—C18X—C17X	1(3)
C14—C13—C18—C17	0.6 (9)	O2X—C13X—C18X—C17X	−176.9 (18)
O2—C13—C18—C19	−2.5 (9)	C14X—C13X—C18X—C19X	−176 (2)
C14—C13—C18—C19	180.0 (5)	O2X—C13X—C18X—C19X	6(3)
C16—C17—C18—C13	−0.5 (7)	C16X—C17X—C18X—C13X	−1(3)
C16—C17—C18—C19	−179.9 (3)	C16X—C17X—C18X—C19X	175.9 (18)
C12—C11—C19—O6	−173.1 (3)	C12X—C11X—C19X—O6X	168 (2)
C10—C11—C19—O6	2.2 (7)	C10X—C11X—C19X—O6X	−1(3)
C12—C11—C19—C18	8.2 (6)	C12X—C11X—C19X—C18X	−10 (3)
C10—C11—C19—C18	−176.5 (4)	C10X—C11X—C19X—C18X	−178.7 (17)
C13—C18—C19—O6	179.0 (5)	C13X—C18X—C19X—C11X	1(3)
C17—C18—C19—O6	−1.6 (5)	C17X—C18X—C19X—C11X	−176 (2)
C13—C18—C19—C11	−2.2 (6)	C13X—C18X—C19X—O6X	−177 (2)
C17—C18—C19—C11	177.2 (4)	C17X—C18X—C19X—O6X	6(3)
C9—C10—C20—C21	154.2 (10)	C11X—C10X—C20X—C21X	−33 (11)
C11—C10—C20—C21	20.1 (15)	C9X—C10X—C20X—C21X	−165 (8)
C9—C10—C20—C23	−36.7 (13)	C11X—C10X—C20X—C23X	156 (7)
C11—C10—C20—C23	−170.8 (9)	C9X—C10X—C20X—C23X	24 (10)
C23—C20—C21—S1	0.5 (13)	C23X—C20X—C21X—S1X	−3(9)
C10—C20—C21—S1	170.7 (9)	C10X—C20X—C21X—S1X	−174 (7)
C22—S1—C21—C20	−1.0 (9)	C22X—S1X—C21X—C20X	14 (5)
C21—S1—C22—C23	1.3 (4)	C21X—S1X—C22X—C23X	−21 (3)
S1—C22—C23—C20	−1.3 (8)	S1X—C22X—C23X—C20X	24 (7)
C21—C20—C23—C22	0.6 (13)	C21X—C20X—C23X—C22X	−15 (10)
C10—C20—C23—C22	−169.9 (8)	C10X—C20X—C23X—C22X	157 (6)

### Hydrogen-bond geometry (Å, °)

**Table:**

*D*—H···*A*	*D*—H	H···*A*	*D*···*A*	*D*—H···*A*
O3—H3B···O4	0.82	1.89	2.7049 (19)	180
O6—H6B···O5	0.82	1.79	2.612 (2)	179
C21—H21A···O4^i^	0.93	2.56	3.475 (8)	168
O3X—H3XB···O4X	0.82	1.86	2.682 (15)	178
O6X—H6XB···O5X	0.82	1.69	2.49 (2)	168
C23X—H23B···O3X^i^	0.93	2.53	3.31 (5)	142

Symmetry codes: (i) −*x*+2, *y*−1/2, −*z*+3/2.
